# Monitoring HIV and AIDS Related Policy Reforms: A Road Map to Strengthen Policy Monitoring and Implementation in PEPFAR Partner Countries

**DOI:** 10.1371/journal.pone.0146720

**Published:** 2016-02-25

**Authors:** Jeffrey Lane, Andre Verani, Mai Hijazi, Erin Hurley, Amy Hagopian, Nicole Judice, Ron MacInnis, Sallie Sanford, Sarah Zelek, Aaron Katz

**Affiliations:** 1 Department of Global Health, University of Washington, Seattle, Washington, United States of America; 2 School of Law, University of Washington, Seattle, Washington, United States of America; 3 Foster Pepper, PLLC, Seattle, Washington, United States of America; 4 Division of Global HIV/AIDS, Center for Global Health, Centers for Disease Control and Prevention, Atlanta, Georgia, United States of America; 5 United States Agency for International Development, Washington, District of Columbia, United States of America; 6 Health Policy Project, Futures Group, Washington, District of Columbia, United States of America; University of Athens, Medical School, GREECE

## Abstract

Achieving an AIDS-free generation will require the adoption and implementation of critical health policy reforms. However, countries with high HIV burden often have low policy development, advocacy, and monitoring capacity. This lack of capacity may be a significant barrier to achieving the AIDS-free generation goals. This manuscript describes the increased focus on policy development and implementation by the United States President’s Emergency Plan for AIDS Relief (PEPFAR). It evaluates the curriculum and learning modalities used for two regional policy capacity building workshops organized around the PEPFAR Partnership Framework agreements and the *Road Map for Monitoring and Implementing Policy Reforms*. A total of 64 participants representing the U.S. Government, partner country governments, and civil society organizations attended the workshops. On average, participants responded that their policy monitoring skills improved and that they felt they were better prepared to monitor policy reforms three months after the workshop. When followed-up regarding utilization of the *Road Map* action plan, responses were mixed. Reasons cited for not making progress included an inability to meet or a lack of time, personnel, or governmental support. This lack of progress may point to a need for building policy monitoring systems in high HIV burden countries. Because the success of policy reforms cannot be measured by the mere adoption of written policy documents, monitoring the implementation of policy reforms and evaluating their public health impact is essential. In many high HIV burden countries, policy development and monitoring capacity remains weak. This lack of capacity could hinder efforts to achieve the ambitious AIDS-free generation treatment, care and prevention goals. The *Road Map* appears to be a useful tool for strengthening these critical capacities.

## Introduction

On February 12, 2013, President Obama proclaimed to a joint session of the U.S. Congress that the United States would help realize “the promise of an AIDS-free generation, which is within our reach.” [1] Strong legal, regulatory, and policy frameworks in partner countries are key to achieving an AIDS-free generation. The PEPFAR Blueprint for an AIDS-free generation highlights the importance of laws, regulations, and policies as barriers and facilitators to an effective HIV response. Barriers include laws that deny services to key populations such as Men who have Sex with Men (MSM), and facilitators include policies that authorize task shifting for increased coverage of Anti-Retroviral Therapy (ART)[2–3]

Soon after the 2008 reauthorization of the United States President’s Emergency Plan for AIDS Relief (PEPFAR) (P.L. 110–293), partner countries entered into non-binding Partnership Framework agreements with the U.S. Government that listed mutual aims and activities for HIV service delivery as well as HIV-related policy reforms to be pursued during the term of the agreements. More recently, the 2013 PEPFAR Stewardship and Oversight Act required expanded annual reporting to Congress including on Partnership Framework agreements (P.L. 113–56). Many of the policy reforms in the Partnership Frameworks are the same reforms identified as critical to achieving an AIDS-free generation [2]. To help build PEPFAR partner countries’ policy development and monitoring capacity in the context of the Partnership Frameworks, an interagency project involving the United States Centers for Disease Control and Prevention (CDC), United States Agency for International Development (USAID), Health Policy Project and University of Washington developed a capacity building tool known as the *Road Map for Monitoring and Implementing Policy Reforms* and used the *Road Map* at a series of workshops attended by multi-sectoral teams from countries with high HIV burdens. This manuscript describes the *Road Map* and workshop curriculum, analyzes the results of an evaluation of both, and discusses the importance of strengthening policymaking and implementation capacity as a critical step for realizing the promise of an AIDS-free generation.

### The Evolution of the Global Policy Response to HIV/AIDS

Public policy decisions have played a prominent role in the history of HIV/AIDS. Following the pandemic’s emergence, country governments struggled with how to appropriately respond to this new threat. For example, in the late 1990s South Africa debated whether the government should finance access to antiretrovirals (ARVs) [4]. Lost benefits due to limited antiretroviral drug use in South Africa have been estimated at 330,000 lives not saved between 2000 and 2005[5]. In 2001, a senior government official in the United States publicly questioned whether ARV therapy could be implemented successfully in resource poor settings [6]. Some governments responded quickly to address the advent of HIV/AIDS, such as Senegal, which has maintained one of the lowest HIV rates in sub-Saharan Africa [7–8]. Experts would later acknowledge that the delay in effective policy responses contributed to the epidemic’s growth and human cost [9]. By 2000 more than 36 million people were infected, a 50% increase from a decade earlier [10].

Around the turn of the millennium the global HIV policy dynamic began to shift. In 1999, governments in sub-Saharan Africa began to declare HIV/AIDS national emergencies [11]. In 2002, the international community launched the Global Fund to Fight AIDS, Tuberculosis and Malaria (Global Fund) to coordinate donor funds. The following year, the U.S. Government launched PEPFAR, which would grow into the largest financial commitment by a nation to globally combat a single disease in history [12]. In 2003, following significant advocacy efforts by civil society, South Africa, the country with the highest number of people living with HIV [13], adopted a plan to publicly fund antiretroviral therapy [4]. Around this same time, policymakers in other high HIV burden countries began to shift policy toward aggressively combating HIV/AIDS [14], including adopting national AIDS strategic plans and related policies [15].

### PEPFAR’s Added Focus on Policy Reform

The U.S. Leadership Against HIV/AIDS, Tuberculosis, and Malaria Act of 2003 (P.L. 108–25) launched PEPFAR in 2003 and authorized approximately $15 billion dollars over a 5-year period for combating HIV, tuberculosis and malaria, with the vast majority of the funding designated for HIV/AIDS. PEPFAR initially spent most of its HIV/AIDS funding in 15 “focus countries” located mostly in sub-Saharan Africa.

As its name suggests, PEPFAR was initially designed as an emergency response. The year the U.S. Government established PEPFAR, only 7% of people in the world in need of treatment for HIV actually received it [16]. To close this gap, PEPFAR emphasized direct service delivery, often partnering with international organizations to establish or expand parallel delivery systems–that is, separate from local public systems. However, PEPFAR also supported HIV service scale-up in public facilities. Using this direct service delivery approach, PEPFAR rapidly expanded the number of people receiving ARVs, providing treatment to 1.45 million people by September 2007, including 1.33 million in sub-Saharan Africa [17]. While this approach served to scale up HIV service delivery, evidence suggests mixed effects on health systems overall [18–21].

In 2008, the Tom Lantos and Henry J. Hyde United States Global Leadership Against HIV/AIDS, Tuberculosis, and Malaria Reauthorization Act of 2008 (P.L. 110–293) reauthorized PEPFAR, increasing its authorized though not appropriated funding for 2009–2013 to $39 billion. The United States Congress ultimately appropriated over $33 billion for this period [22]. The 2008 PEPFAR reauthorization broadened its focus to include strengthening partner country public health systems while maintaining ambitious direct service delivery targets. This law authorized PEPFAR to enter into publicly available, multi-year Partnership Framework agreements with partner country governments.

By June 2012, 22 Partnership Frameworks had been negotiated and signed with 16 countries in Africa (Angola, Botswana, Democratic Republic of the Congo, Ethiopia, Ghana, Kenya, Lesotho, Malawi, Mozambique, Namibia, Nigeria, Rwanda, South Africa, Swaziland, Tanzania, Zambia), Haiti, Dominican Republic, Vietnam, and Ukraine and with several countries from two regions, Central America and the Caribbean. The Partnership Frameworks are publicly available at www.pepfar.gov. Many countries also worked with PEPFAR to develop more detailed Partnership Framework Implementation Plans (PFIPs), which contained greater detail regarding implementation of the Partnership Frameworks. The Partnership Framework negotiating and signature process provided senior United States Government representatives an opportunity to engage with senior Ministry of Health and other executive branch representatives from PEPFAR partner countries. For example, the South African Partnership Framework was signed by U.S. Secretary of State (Hillary R. Clinton) and the Republic of South Africa Minister of International Relations and Cooperation (Maite Nkoana-Mashabane). South Africa’s PFIP was subsequently signed by the U.S. Ambassador to the Republic of South Africa and the Minister of Health of the Republic of South Africa.

The Partnership Framework guidance called for the U.S. Government and partner governments to identify key HIV-related policy reforms in the following areas: human resources for health; gender; children; counseling and testing; access to high quality, low cost medications; stigma and discrimination; and strengthening multi-sectoral linkages [23]. Consistent with this guidance, each Partnership Framework contains policy reforms to be pursued by PEPFAR-funded countries. The policy reforms cover a wide range of topics from clinical guidelines to gender-based violence and orphans and vulnerable children. Some examples of policy reforms planned in the Partnership Frameworks include: development of national health financing plan in Haiti; introduction and passage of gender-based violence bill in Zambia; development and adoption of task-shifting policy in Lesotho; development of stigma and discrimination policy in Ghana; implementation of pharmaceutical logistic management plan in Ethiopia; and development of male circumcision guidelines in Rwanda.

### Planned Policy Reforms in the Partnership Frameworks

To assess the prominence and role of policy in the Partnership Frameworks, we conducted a global analysis of the planned policy reforms listed in the Partnership Frameworks. For the purpose of our review, we defined “policy reform” as a government course of action codified in an official government document (e.g., legislation, regulations, guidelines, strategic plans, or budgets) and identified for some type of action during the term of the Partnership Framework (e.g., revision, development, implementation). These reforms are referred to by various names across partner countries, but have the ultimate goal of improving health outcomes.

The 22 Partnership Frameworks contain 255 policy reforms and 589 distinct activities relating to these policy reforms. The number of policy reforms per Partnership Framework ranged from zero to 28, with a median of nine policy reforms per Partnership Framework. The Partnership Framework with zero policy reforms was South Africa, because its general references to policy reform did not meet our more specific inclusion criteria. However, the South Africa PFIP, which is publicly available online and more detailed than the Partnership Framework [24], identifies a number of policy reforms. The most common program areas addressed by policy reforms are Human Resources for Health, Clinical Services and Other Prevention. Table 1 shows a breakdown of how policy reforms span the different PEPFAR program areas. The complete dataset is provided as a supporting information file (S1 File).

**Table 1:** Policy Reforms planned in 22 PEPFAR Partnership Frameworks.

Program Area	Total No. of Policy Reforms	Develop New Policy	Revise Existing Policy	Implement Existing Policy
Human Resource for Health	32	15	6	9
Clinical Services	26	11	6	9
Other Prevention	21	12	2	5
HIV Testing and Counseling	17	10	3	3
Strategic Information	14	10	1	3
Orphans & Vulnerable Children (OVC)	14	7	2	5
Laboratory	13	10	1	2
Commodity Procurement & Supply Chain	11	5	4	2
Voluntary Medical Male Circumcision	11	9	2	0
Tuberculosis (TB) / HIV	8	6	0	2
Prevention of Mother-to-Child Transmission of HIV	8	4	3	1
Children (not OVC)	8	4	0	3
Stigma & Discrimination	8	4	0	3
Multi-sectoral Linkages	7	4	0	2
Organization of Services	7	5	0	2
Gender	7	4	0	2
Access to Drugs	4	1	1	1
Other	39	21	10	8
**Total**	**255**	**142**	**41**	**62**

**Fig 1:**
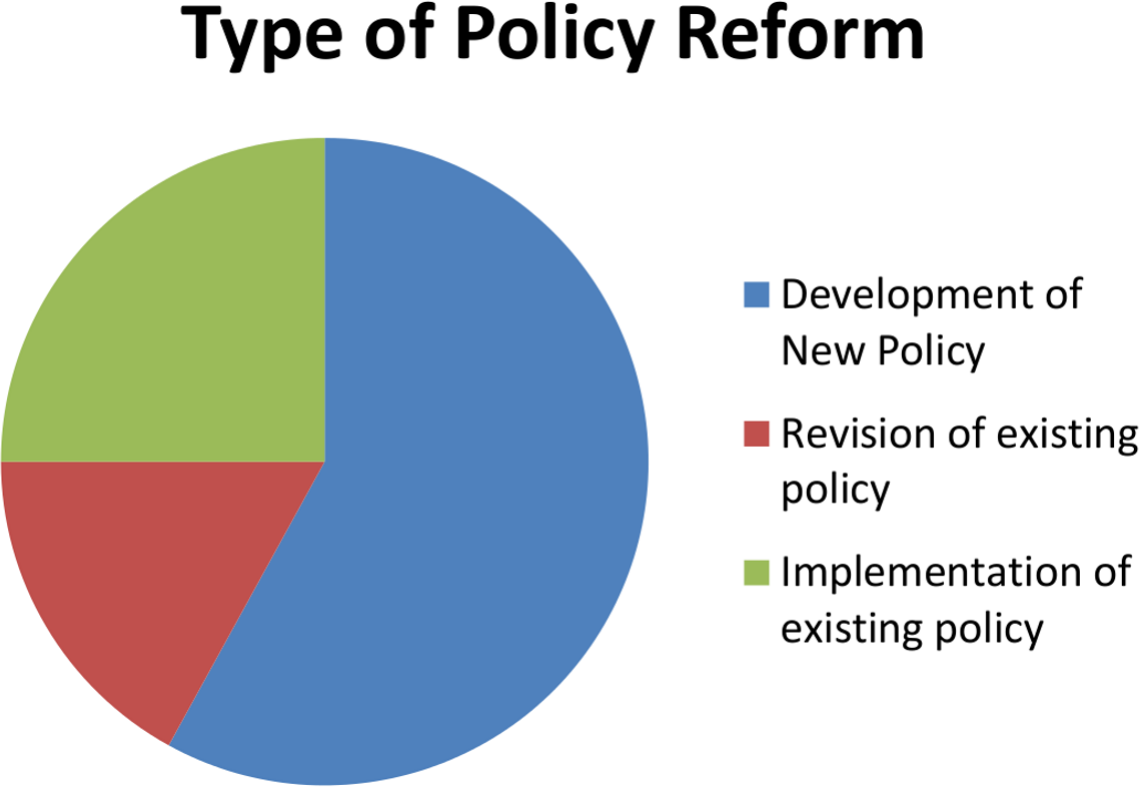
Type of Policy Reform.

Of the 245 policy reforms providing sufficient detail to code, 58% called for developing a new policy; 17% for revising an existing policy; and 25% for implementing an existing policy (Fig 1). We observed that the policy reform agendas in the PFIPs may have differed somewhat from the Partnership Frameworks, and in some cases included more ambitious policy reform agendas. We did not include PFIPs in our analysis, because they are not all public documents.

Together, PEPFAR and partner countries are monitoring policy reforms identified in the Partnership Frameworks. PEPFAR has historically focused monitoring efforts on service delivery targets, but starting in 2010, PEPFAR country teams also began to report progress on the policy objectives identified in the Partnership Frameworks by using a six stage policy monitoring framework [25]:

- Stage 1 –Identification of baseline policy issues
- Stage 2 –Engagement of stakeholders in developing common policy agenda
- Stage 3 –Development of policy
- Stage 4 –Official government endorsement (i.e., passage) of policy
- Stage 5 –Implementation of policy
- Stage 6 –Evaluation of policy implementation

Similar multi-stage policy development and adoption frameworks, referred to as the heuristic stages, have been discussed at length in the public policy literature [26–27]. The policy reports illustrate that some countries have successfully moved some policy reforms along the six stage framework during the term of their Partnership Framework. Although a thorough analysis of actual policy reform progress is beyond the scope of the present paper and is being conducted separately, examples of progress include Mozambique, a country with one of the lowest doctor-to-patient ratios in the world [28], adopting a policy allowing for ART task-sharing with nurses [29], and Lesotho passing a Medicines Control Act to ensure the quality of medicines including antiretrovirals. Countries are also often quite successful at updating treatment guidelines to reflect WHO recommendations. A 2010 WHO survey conducted on ARV use in low and middle-income countries found that the WHO’s 2006 treatment recommendations had largely been adopted and implemented by national HIV programs and that many countries were in the process of reviewing and updating ARV treatment guidelines [30–31]. These success stories illustrate that some countries are making meaningful progress in reforming policies critical to realizing an AIDS-free generation. [32] However, in many high HIV burden countries little progress is being made on many other critical policy reforms, including policies addressing gender-based vulnerabilities, key populations, orphans and vulnerable children, and financing [33].

### Policy Reforms for an AIDS-Free Generation

The variety of policy reforms needed to achieve an AIDS-free generation will be unique to each country, yet some key evidence-based policy reforms relevant in many countries include: revising antiretroviral initiation guidelines to start treatment earlier in the disease progression; implementing programs to prevent mother-to-child transmission and to keep all HIV+ women alive and healthy (e.g., PMTCT Option B+); increasing national budget expenditures for HIV programs; ensuring that marginalized populations have access to care and treatment services; and revising human resources laws and policies to allow more trained health care workers to prescribe antiretroviral therapy. These policy reforms have been shown to improve the scale and effectiveness of national HIV programs [2]. Monitoring these reforms, however, can be challenging. Policy reforms in all countries are memorialized in a range of documents (e.g., legislation, operational plans, strategic frameworks, guidelines, and budgets) and span a range of sectors (e.g., human resources for health, financing, laboratory, supply chain management, procurement, quality assurance, health information technology and many others). This multi-sectoral context makes collecting reliable and actionable data difficult, as each sector often has its own reporting mechanisms, information needs, and monitoring approaches.

## Methods

To strengthen the capacity of stakeholders in PEPFAR supported countries to monitor and implement HIV-related policy reforms, PEPFAR funded two regional workshops in August and September 2012 organized around the *Road Map for Monitoring and Implementing Policy Reforms*. Thirty-three participants from Zambia, Malawi, Tanzania, Kenya, and Nigeria attended the Africa regional workshop. Thirty-one participants from Haiti, the Dominican Republic and several other nations from the Caribbean and Central America attended the Americas regional workshop. Most attending country teams were multi-sectoral, with representatives from national government (19 total), civil society (10 total), multilateral organizations (3 total) and U.S. Government in-country offices (32 total). Country government attendees represented a variety of governmental agencies, including ministries of health, finance, and development, and national AIDS commissions.

Facilitators from the USAID, CDC, Health Policy Project and University of Washington developed a workshop curriculum to achieve the following purposes: facilitate international sharing of progress and challenges in monitoring policy reforms listed in Partnership Frameworks and Partnership Framework Implementation Plans; review policy monitoring methodologies and tools; and promote development of draft policy monitoring *Road Maps* by each attending country team. The expected outcomes of the workshop included: improved awareness of progress and challenges across countries in policy reform monitoring; increased knowledge of policy monitoring methods and tools; and strengthened in-country capacity to monitor and implement policy reforms. The workshops used four primary learning methodologies: (1) country team presentations; (2) didactic sessions; (3) participant panels organized by sector; and (4) structured group exercises organized around the *Road Map for Implementing and Monitoring Policy Reforms*.

Country teams opened the workshops with presentations on the current status of their policy development and monitoring activities. These presentations facilitated collaborative learning concerning the successes and struggles of other countries in their region around policy development, implementation and monitoring.

Workshop facilitators led didactic sessions on a range of issues to ensure that all participants had basic knowledge regarding the fundamentals of policy development, implementation, and monitoring. Didactic session topics included policy development, policy communications, legal mechanisms for addressing HIV/AIDS, and policy monitoring methods. Whenever possible, session moderators pre-arranged for workshop participants with relevant experiences and/or expertise to co-lead part of these didactic sessions.

The workshops also included a series of moderated panel sessions organized by sector (i.e., civil society, U.S. government in-country offices, and national government). These panels offered a unique opportunity to highlight differing perspectives, roles and attitudes of key stakeholder groups such as the healthy conflict that can arise between civil society organizations and national government agencies.

The core of the workshop was a series of team exercises built around the *Road Map for Monitoring and Implementing Policy Reforms*. The *Road Map* was developed jointly by CDC, USAID, Health Policy Project and University of Washington based on a literature review and prior technical assistance experience. The *Road Map* contains a series of eight exercises designed to prepare each national team to actively pursue policy monitoring activities in their countries. The complete *Road Map* template is provided as a supporting information file (S2 File). A brief description of each section of the *Road Map* follows:

1. *Current Status of Implementing Policy Interventions*: The first step in completing the *Road Map* is to assess the current status of key policy reforms.
2. *Priority Setting Worksheet*: Teams apply this priority-setting sheet to determine which policy interventions they wish to address throughout the course of this workshop.
3. *Policy Monitoring Stakeholder Analysis*: The Stakeholder Analysis Matrix can be applied to each selected policy intervention to identify key stakeholders. The matrix also assists countries in thinking through each stakeholder’s potential role in the policy process, level of commitment, available resources and constraints.
4. *Pathway to Policy Change*: The Pathway approach is based on the theory of change model and allows teams to define the steps involved in adopting a policy reform, so that they can identify specific interventions/actions and the process and output indicators that would be used to monitor the policy process.
5. *Policy Monitoring Logic Model*: The Logic Model captures the information needed to monitor policy implementation, including data sources and key assumptions.
6. *Action Plan for Implementing and Monitoring Individual Policy Interventions*: Teams use the tools and questions provided to develop a monitoring plan for a specific policy intervention. The plan can help identify additional resources, structures, processes, or data needed to keep track of progress.
7. *Post-Workshop Country Plan for Monitoring Policy Interventions*: Teams identify important post-workshop action steps to establish or strengthen a policy monitoring system.
8. *Self-Assessment of Country Policy Monitoring*: These questions are discussed throughout the course of the workshop and help guide the development of action plans.

The *Road Map* was designed to build foundational competencies to engage in policy development and monitoring, assess existing policy monitoring systems in the team’s country, and identify next steps to strengthen or establish policy monitoring systems.

The project team evaluated the usefulness of the workshops and the *Road Map*. The evaluation plan consisted of session evaluations, end-of-day evaluations, feedback discussion groups, and a follow-up evaluation at three months following each workshop. Session evaluations were completed twice per day during the first workshop (before lunch and at end of day), but only once per day at the second workshop (end of day). The follow-up evaluations were conducted using an online survey sent to participants approximately three months after the completion of the workshop they attended. The evaluation surveys included both open-ended qualitative and quantitative questions ranking responses on a scale of one to five.

## Results

A total of 64 participants (33 in Africa workshop, 31 in Americas workshop) representing the U.S. Government, partner country governments, and civil society organizations from seven countries and two regions attended the two workshops. Participants were asked at the start and end of the workshops to rank their policy monitoring skills on a scale of 1 to 5, with 1 being poor and 5 being excellent. On average, participants responded that their policy monitoring skills increased from 2.9 out of 5 before the workshop to 4.0 out of 5 after participating in the workshop. Attendees of the Africa workshop reported an increase from 3.0 (average) to 4.1 (good). Attendees of the Americas workshop reported an increase from 2.8 (average) to 3.9 (good).

**Fig 2:**
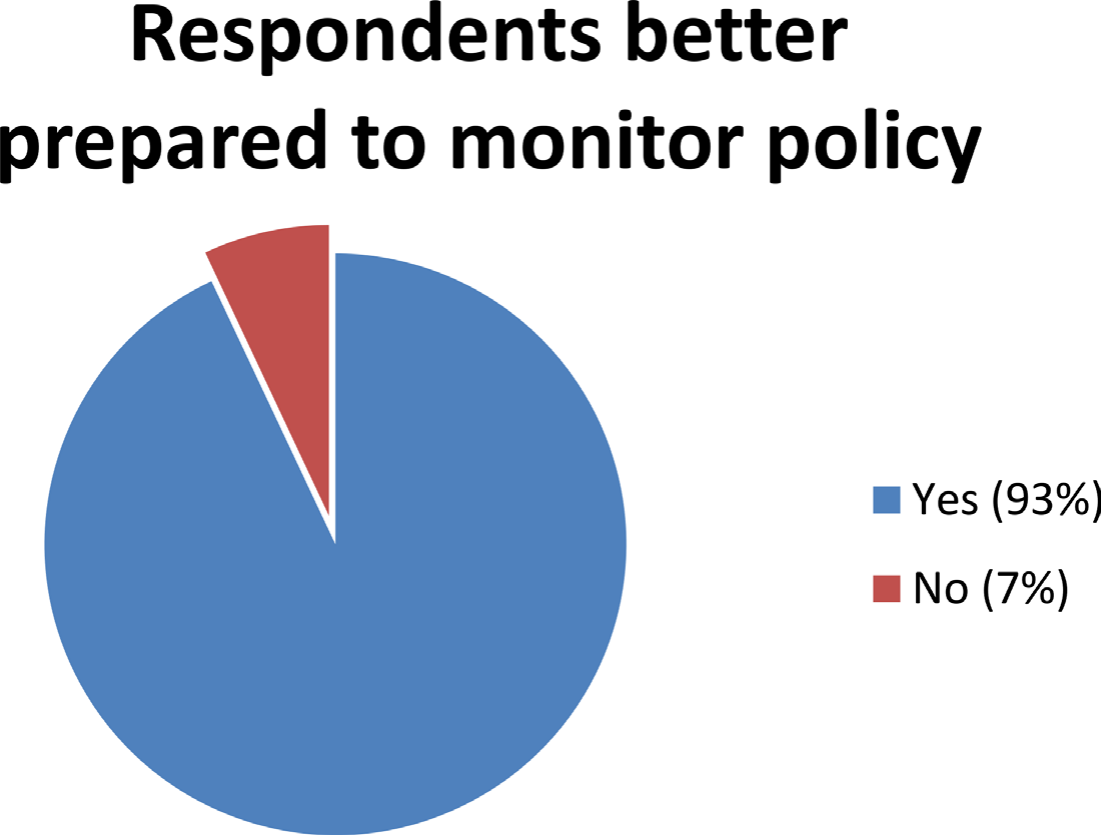
Respondents better prepared to monitor policy.

The three month follow-up evaluation found a majority of respondents considered the workshop useful. Eighty-five percent of respondents from the Africa workshop and 100% of respondents from the Americas workshop said they felt they were better prepared to monitor policy reforms after the workshop (Fig 2).

Also, on a scale of 1–5 (1 being “least,” 5 being “most”), 63% of respondents rated the usefulness of the workshop as 4 out of 5. The Africa workshop respondents had an average rating of 3.8, while the Americas respondents’ average rating was slightly higher at 4.0. A limitation is the low overall response rate for the three month follow-up evaluation (42.2%).

The workshop evaluations illustrated that the diverse learning modalities (panels, didactic, group exercises, and country presentations) helped keep participants engaged and facilitated opportunities for cross-country learning. The sector-based panels were rated highest out of all of the different session modalities (4.2 out of 5).

The evaluations completed at the workshops evaluated the *Road Map* sessions as 4.0 out of a possible 5 points. The *Road Map* session with the highest score was the Post-Workshop Plan, during which teams agreed on specific action steps to pursue in their countries.

The action plans developed as part of the *Road Map* proved to be quite ambitious. A number of teams decided to establish a policy monitoring committee to coordinate the national policy monitoring process. Other interesting next steps included: reducing the number of indicators to a manageable level; harmonizing policy monitoring with national HIV/AIDS Monitoring and Evaluation (M&E) plan; and scheduling meetings with potential policy champions for priority reforms.

The three month evaluation illustrated that while some countries made quick progress in implementing elements of their action plan, other countries struggled to gain traction when confronting the realities of competing priorities and limited resources. When participants from both workshops were asked if they or their team had further developed the action plan started at the workshop, 10 of the 27 respondents responded ‘yes’. For the Africa workshop 23% of the respondents said yes. For the Americas workshop, 50% of the respondents answered yes. Reasons cited for not making progress on their action plan included an inability to meet or a lack of time, personnel, or governmental support. Participants requested more follow-up, real examples of policy monitoring indicators and best practices, country specific M&E training, and the development of a participant listserv. Efforts are ongoing to address participants’ requests for further information and training on policy M&E.

Balanced teams with multi-sectoral representation from civil society, U.S. government in-country staff, and country government sectors appeared important to the success of the workshop. Teams with better multi-sectoral representation were better able to address a wider array of barriers to policy reform. Representation from ministries of finance was especially helpful in addressing the realities of trying to finance ambitious policy reform goals.

During the workshop it also became clear that some representatives from civil society and country government were not familiar with the policy reform agendas in the Partnership Frameworks or the PFIPs. The lack of familiarity with the PFIPs may have been partially due to those documents not being as readily available as the Partnership Framework. Increased difficulty accessing PFIPs also seemed to undermine their value as a policy and planning tool, in contrast to the publicly available Partnership Frameworks.

## Discussion

To achieve an AIDS-free generation, countries with high HIV burdens will need to dramatically scale up and sustain existing HIV-related programs, within supportive legal, regulatory, and policy frameworks. In contrast to program implementation monitoring which has attracted a great deal of analysis and attention in the literature, systematic monitoring of policy development and implementation remains a novel concept to many public health practitioners. Additionally, policy reform remains an abstract and amorphous concept to many stakeholders who are essential to successfully adopting and implementing these reforms. Many important stakeholders–often including the public–are skeptical about whether policy-level decisions bear any benefit to real people. Moreover, doctors, nurses, public health workers, hospital administrators, and business leaders often see policy reform as an activity of legislators, members of parliament, or senior ministry staff to which they have no access. Successful health policy reform, however, benefits from a transparent process with broad stakeholder involvement, stakeholders that understand the policy process and actively seek a role in shaping policy decision-making and implementation. Strengthening the capacity of key sectors in PEPFAR partner countries to monitor policy reform and implementation is thus a critical step in achieving the AIDS-free generation targets.

This manuscript describes one approach to strengthen the capacity of multi-sectoral stakeholders to engage in the implementation and monitoring of key health policy reforms. This intervention was structured as a 4-day workshop, yet the *Road Map* could be utilized in other formats. For example, *Road Map* group exercises could be spread out over multiple weeks or discrete sections of the *Road Map* could be used for specific purposes (e.g., setting policy monitoring priorities).

Limitations of our workshop participant evaluations included a low response rate which hinders generalizability to all workshop participants and possible selection bias as only 9 of the 22 operating units (e.g., PEPFAR country or regional offices) with signed Partnership Frameworks were able to participate in the policy monitoring capacity building workshops.

Our project also called attention to a need for building policy monitoring systems in PEPFAR partner countries. Most countries lack a unit with the personnel and technical skills needed to monitor progress on key policy reforms. In some countries, multi-stakeholder technical working groups that advise government on HIV policy issues monitor progress on developing and implementing policies specific to their technical area, but cross-sector policy monitoring bodies are quite rare. As a result, a number of teams attending the policy monitoring workshops planned to establish such a body. Technical working groups may continue to play a key role in monitoring specific policies, but cross-sector monitoring bodies will be able to assess whether a country’s policies are progressing in a direction that will ultimately provide universal access to quality prevention, care, and treatment programs.

These national policy monitoring bodies will likely need to set priorities for monitoring. The Partnership Frameworks often listed dozens of planned policy reforms and activities, overwhelming most monitoring and evaluation systems. To improve the quality of policy reform monitoring, these bodies should identify a smaller number of important reforms and monitor them closely. Alternatively, countries could develop two or three tiers of priority policy reforms as was done in the Malawi Partnership Framework. This would allow countries to develop a robust list, but still allow them to efficiently allocate time and financial resources toward the highest priority reforms. The need to prioritize became obvious to some workshop participants while completing the *Road Map*.

Agreements between donor and partner governments (e.g., PEPFAR Partnership Frameworks, Millennium Challenge Corporation Compacts) can play an important role in providing an agreed and written multi-year plan to achieve common goals, including policy reforms [34]. However, because the success of policy reforms cannot be measured by the mere adoption of written policy documents, monitoring the implementation of policy reforms and evaluating their public health impact is essential. To efficiently monitor policy implementation, countries should seek to integrate policy monitoring indicators with existing program monitoring and evaluation systems. This may be done by pairing high level policy reform indicators with facility and patient level program indicators, thus assessing national policy changes alongside potentially associated changes in public health practice. For example, policy monitoring indicators for developing and adopting a PMTCT Option B+ policy can be linked with service delivery indicators, such as percentage of pregnant women initiated on ART for life, to assess whether the policy reform impacted service delivery [35]. These service delivery indicators might also help identify policy implementation barriers. The Policy Monitoring Logic Model and Action Plan included in the *Road Map* can be used to identify and document linkages between policy reform and programmatic indicators. Linking policy indicators with service provision indicators can assist in identifying upstream causes for underperformance and could increase the uptake of policy monitoring data by strategic information and public health surveillance teams. Combining policy and service provision monitoring systems would also help achieve the “Three Ones” principle of one HIV/AIDS strategic plan, one HIV/AIDS coordinating body, and one monitoring and evaluation system.

Improving the quality and transparency of policy monitoring data is critical for tracking progress toward the AIDS-free generation targets and ensuring that policymakers have accurate information to inform programmatic and policy decision-making. Policymakers can use this data to check national progress and, if necessary, make policy course corrections. Making policy monitoring data transparent should increase its potential impact. If shared broadly, policy monitoring data could become a critical tool for civil society engagement and advocacy.

## Conclusion

The AIDS-free generation agenda reflects the growing evidence showing the effectiveness of existing HIV/AIDS service delivery programs and growing confidence in the ability of high HIV burden countries to take the lead in managing and financing their national HIV response. Yet, in many of these countries, policy development, advocacy, and monitoring capacity in key sectors remains weak, which could undermine efforts to achieve and sustain the ambitious AIDS-free generation treatment, care and prevention goals. The *Road Map for Monitoring and Implementing Policy Reforms* appears to be a useful tool for strengthening these critical capacities, to move one step closer to an AIDS-free generation.

## Supporting Information

S1 File Policy Reforms in the Partnership Frameworks. (XLS)

S2 File Road Map for Monitoring and Implementing Policy Reforms. (DOCX)

S3 File Workshop Evaluation Module 1A. (DOCX)

S4 File Workshop Evaluation Module 1B. (DOCX)

S5 File Workshop Evaluation Module 1C. (DOCX)

S6 File Workshop Evaluation Module 3-Month Follow-up. (DOCX)
