# Supplementary material for: Monitoring HIV and AIDS Related Policy Reforms: A Road Map to Strengthen Policy Monitoring and Implementation in PEPFAR Partner Countries
Source: PLoS One. 2016 Feb 25;11(2):e0146720. doi: 10.1371/journal.pone.0146720 (PMC4767332; doi:10.1371/journal.pone.0146720)
Supplement: S2 File — (DOCX) [file pone.0146720.s002.docx]

**S2 File**

**Road Map for Monitoring and Implementing Policy Reforms**

# Current Status of Implementing Policy Interventions—Monitoring the Policy Process

The first step in completing the *Road Map* is to assess the current status of the policy intervention by documenting what has already been accomplished according to the six policy stages.

- 1. Which policy interventions are priorities for your country? Are any of these policy interventions interlinked?
  2. Which policy interventions are on track and why (that is, what factors have helped to move these along?)
  3. Which policy interventions are stalled or moving slowly and why?

| **Policy Intervention** | **Responsible Party(ies)** | **Stage 1: Situation Assessment** | **Stage 2: Policy Agenda** | **Stage 3: Develop Policy** | **Stage 4: Endorsement** | **Stage 5: Implementation** | **Stage 6: Evaluation** |
| --- | --- | --- | --- | --- | --- | --- | --- |
| ***Example****: Support Ministry of Medical Services (MOMS) / Ministry of Public Health and Sanitation (MOPHS) to develop and implement incentives / standards of employment for equitable treatment, distribution, & retention of all health workers* |  |  |  |  |  |  |  |
| ***Example****: Enact policy changes for training program of Enrolled Community Nurses, Registered Nurses, and Community Health Extension Workers* |  |  |  |  |  |  |  |
|  |  |  |  |  |  |  |  |

# Priority Setting Worksheet

The below worksheet can be used to analyze which policies should be prioritized for monitoring. Each criterion should be given a letter grade with “A” representing the most ideal for that criterion and “D” representing the least ideal for that criterion. After grading each criterion, your team should enter an overall priority grade and explain why you gave that overall grade.

Before completing the below worksheet, teams should answer the following questions:

1. Are any of the below criteria more important than others? Why?
2. Should any additional criteria be considered when prioritizing policies for monitoring?
3. Has your country already established a list of priority policies?
4. What other stakeholders should be consulted when deciding which policies should be prioritized for monitoring?

| **Policy** | **Cost of Policy Implementation**   - Funding already allocated? | **Likelihood of Political Progress**   - Political window? - Government champion? | **Magnitude of Health Impact**   - Evidence-base? - Scale of intervention? | **Cost of Monitoring**   - Indicators already being tracked? - Could indicators be added to existing M&E system? | **Monitoring Findings Likely to be Used?**   - By civil society? - By policymakers? - By funders? | **Overall Grade**   - Why did you give this grade? |
| --- | --- | --- | --- | --- | --- | --- |
| Enter name of policy in this column | Low Cost = A  Medium Cost = B  Cost Uncertain = C  High Cost = D | Progress Very Likely = A  Progress Likely = B  Progress Uncertain = C  Progress Unlikely = D | High Impact = A  Medium Impact = B  Uncertain Impact= C  Low Impact = D | Low Cost = A  Medium Cost = B  Cost Uncertain = C  High Cost = D | High Likelihood = A  Med. Likelihood = B  Uncertain = C  Low Likelihood = D | High Priority = A  Medium Priority = B  Low Priority = C  Very Low Priority = D |
| 1. |  |  |  |  |  |  |
| 2. |  |  |  |  |  |  |
| 3. |  |  |  |  |  |  |
| 4. |  |  |  |  |  |  |
| 5. |  |  |  |  |  |  |
| 6. |  |  |  |  |  |  |
| 7. |  |  |  |  |  |  |
| 8. |  |  |  |  |  |  |

# Policy Monitoring Stakeholder Analysis

After identifying a key policy intervention, it is important to understand the stakeholders interested in that particular policy intervention. The *Stakeholder Analysis Matrix* can be applied to each selected policy intervention.

- 1. Who are the key stakeholders for your policy interventions? Which technical groups and key stakeholders need to be engaged to help move policy interventions forward?
  2. How are you currently engaging your key stakeholders or how will you engage these groups/stakeholders?
  3. How do you communicate your policy progress on policy development and implementation to these stakeholders?

**Policy Intervention _______________________________________________________________________________________**

**Current Status (Policy Stage) ____________________________________________________________________________­­­___**

| **Name of stakeholder organization, group or individual**  *National, regional or local?* | **Stakeholder description**  *Primary purpose, affiliation, funding* | **Potential role in the policy process**  *Vested interest, role, responsibility* | **Level of knowledge of the issue**  *Specific areas of expertise* | **Level of commitment**  *Support or oppose the activity, to what extent, and why?* | **Available resources**  *Staff, volunteers, money, technology, information, influence* | **Constraints**  *Limitations: need funds to participate, lack of personnel, political or other barriers* |
| --- | --- | --- | --- | --- | --- | --- |
| Government sector | | | | | | |
|  |  |  |  |  |  |  |
|  |  |  |  |  |  |  |
|  |  |  |  |  |  |  |
|  |  |  |  |  |  |  |
|  |  |  |  |  |  |  |
| Political sector | | | | | | |
|  |  |  |  |  |  |  |
|  |  |  |  |  |  |  |
|  |  |  |  |  |  |  |
|  |  |  |  |  |  |  |
| Commercial sector | | | | | | |
|  |  |  |  |  |  |  |
|  |  |  |  |  |  |  |
|  |  |  |  |  |  |  |
|  |  |  |  |  |  |  |
| Non-governmental sector | | | | | | |
|  |  |  |  |  |  |  |
|  |  |  |  |  |  |  |
|  |  |  |  |  |  |  |
|  |  |  |  |  |  |  |
| Other civil society target audiences | | | | | | |
|  |  |  |  |  |  |  |
|  |  |  |  |  |  |  |
|  |  |  |  |  |  |  |
|  |  |  |  |  |  |  |
| International donors | | | | | | |
|  |  |  |  |  |  |  |
|  |  |  |  |  |  |  |
|  |  |  |  |  |  |  |
|  |  |  |  |  |  |  |

# Pathway to Policy Change—Identifying Key Indicators to Monitor Policy Process

Outcome and Desired Impact of the Policy Intervention

*Example Only*

**Ask these questions:**

- *What options/what conditions must exist to implement the policy intervention?*
- *Who has the authority to authorize and/or implement the policy?*
- *What bottlenecks have to be overcome to achieve the policy intervention?*
- *What is the timeframe for reaching a given point?*

Validation of standards among professional unions/associations, CSOs and government

Ministry of Health develops standards through consultations

MOH and Min. of Labor disseminates standards and implementation plan

Validation of standards among professional unions/associations, CSOs and government

Final implementation plan, including specific monitoring indicators, for employment standards

*Policy Intervention*: Develop and implement standards of employment for equitable treatment, distribution and retention of all health workers

# Logic Model

## Policy Monitoring Logic Model (selected policy intervention)

The logic model captures the information needed to monitor and evaluate the policy process and ultimately the success of policy implementation. The policy development and implementation process will feed into the logic model’s process and output indicators.

|  | **Inputs** | **Processes** | **Outputs** | **Outcomes**  (i.e., overall goal or purpose of the policy intervention) | **Impact** |
| --- | --- | --- | --- | --- | --- |
| **Indicators** | *Examples:* funding, staff, material resources | *Examples*: trainings, consultative forum | *Examples*: # of policies and plans developed, implementation plans | *Examples*: health status intervention, improved service quality or effectiveness |  |
| **Data source** |  |  |  |  |  |
| **Evidence and/or Assumptions** |  |  |  |  |  |

# Action Plan for Implementing and Monitoring Individual Policy Interventions

| **Policy Intervention Title:** | | | | | | |
| --- | --- | --- | --- | --- | --- | --- |
| **Policy** **Stage** (as of current date, please provide brief narrative): | | | | | | |
| **Actions/steps** | **Responsible party(ies)** | **Indicator(s)**  **(derived from pathway and logic model)** | **Data source Existing? Frequency of collection & reporting?** | **Interested stakeholders** | **Communication methods**  **What form & frequency are most appropriate for key audience(s)?** | **Timeline** |
| *Example:* Convene consultative forum to discuss evidence base for developing and adopting new employment standards | *Intervention*: |  |  |  |  |  |
|  | *Monitoring*: |  |  |  |  |  |
| *Example:* Develop employment standards policy document | *Intervention*: |  |  |  |  |  |
|  | *Monitoring*: |  |  |  |  |  |
|  | *Intervention*: |  |  |  |  |  |
|  | *Monitoring*: |  |  |  |  |  |
|  | *Intervention*: |  |  |  |  |  |
|  | *Monitoring*: |  |  |  |  |  |
|  | *Intervention*: |  |  |  |  |  |
|  | *Monitoring*: |  |  |  |  |  |
|  | *Intervention*: |  |  |  |  |  |
|  | *Monitoring*: |  |  |  |  |  |
|  | *Intervention*: |  |  |  |  |  |
|  | *Monitoring*: |  |  |  |  |  |
|  | *Intervention*: |  |  |  |  |  |
|  | *Monitoring*: |  |  |  |  |  |

# Post-Workshop Country Plan for Monitoring Policy Interventions

**This plan will reflect immediate commitments of the team to strengthen monitoring of policy interventions*.

| **Commitments**  **Immediate actions** | **Who?** | **When?** |
| --- | --- | --- |
| *Example 1:* Convene a policy monitoring committee  *Example 2:* Identify coordinator for policy monitoring committee  *Example 3:* Finalize action plan for priority policy interventions  *Example 4:* Meet with MOH to discuss the addition of policy monitoring indicators to national HMIS |  |  |
|  |  |  |
|  |  |  |
|  |  |  |
|  |  |  |

# Self-Assessment of Country Policy Monitoring

**1. Policy Interventions**
These questions can be used to discuss Tools A and B

1. Which policy interventions are priorities for your country? Are any of these policy interventions interlinked?
2. Which policy interventions are on track and why (that is, what factors have helped to move these along?)
3. Which policy interventions are stalled or moving slowly and why?
4. Are there any priority HIV policy interventions, which are not included in national policy documents?
5. Which policy intervention(s) does your team wish to address during the duration of this workshop (*please select three of interest and focus on one for the duration of the workshop*)?

**2. Stakeholders**These questions can be used to discuss Tool C

1. Who are the key stakeholders for your policy intervention? Which technical groups and key stakeholders need to be engaged to help move the policy intervention forward?
2. How are you currently engaging your key stakeholders or how will you engage these groups/stakeholders?
3. How do you communicate your policy progress on policy development and implementation to these stakeholders?

**3. Data and evidence**These questions can be used to discuss Tool E

- 1. What indicators do you currently use to monitor policy development and implementation?
  2. What challenges and successes have you encountered relating to these indicators?
  3. What data sources do you use to monitor policy development and implementation?
  4. What challenges and successes have you encountered relating to these data sources?
  5. Are these data used to communicate the importance of policy? Are these data used to inform and overcome policy changes or bottlenecks?

**4. Policy Monitoring Process and Responsibilities**These questions can be used to develop the plans Tool F and G

- 1. Who monitors progress on your policy intervention? Are policy indicators currently monitored in the national HMIS or other information systems?
     1. Are responsibilities for monitoring clear? If not, what would make them clearer?
  2. Would the inclusion of other parties strengthen this process? If so, who?
  3. Does your country have any other health policy monitoring processes in place?
     1. If so, do these other processes overlap with your policy monitoring process(es)? Could these other processes be used to help monitor policy interventions?

**5. Resources/Capacity Needs**

- 1. Who provides or supports technical, staffing and financial resources monitoring policy interventions? Have these resources been adequate?
  2. What tools and technical competencies has your country used to monitor policy development and implementation?
  3. What other tools or technical competencies would strengthen your policy monitoring activities?
