# Supplementary material for: Monitoring HIV and AIDS Related Policy Reforms: A Road Map to Strengthen Policy Monitoring and Implementation in PEPFAR Partner Countries
Source: PLoS One. 2016 Feb 25;11(2):e0146720. doi: 10.1371/journal.pone.0146720 (PMC4767332; doi:10.1371/journal.pone.0146720)
Supplement: S3 File — (DOCX) [file pone.0146720.s003.docx]

**DAY 1, SESSION 1: Workshop Objectives – 9:30–10:00**

*On a scale of 1-5, please evaluate this session by circling your answer*

Usefulness of the material

Poor…………………..…Fair……………..…...…Average……………….…...Good…………..…Excellent

1 2 3 4 5

Organization of session

Poor…………………..…Fair……………..…...…Average……………….…...Good…………..…Excellent

1 2 3 4 5

Clarity of presentation

Poor…………………..…Fair……………..…...…Average……………….…...Good…………..…Excellent

1 2 3 4 5

Would you recommend that this session be included in future PEPFAR policy monitoring workshops with other countries? Check one.

☐ Yes ☐ No ☐ Unsure

Comments (optional):

PF/PFIP Workshop – (1A)

**DAY 1, SESSION 2: *Why policy?* – 10:00–10:30**

*On a scale of 1-5, please evaluate this session by circling your answer*

Usefulness of the material

Poor…………………..…Fair……………..…...…Average……………….…...Good…………..…Excellent

1 2 3 4 5

Organization of session

Poor…………………..…Fair……………..…...…Average……………….…...Good…………..…Excellent

1 2 3 4 5

Clarity of presentation

Poor…………………..…Fair……………..…...…Average……………….…...Good…………..…Excellent

1 2 3 4 5

Would you recommend that this session be included in future PEPFAR policy monitoring workshops with other countries? Check one.

☐ Yes ☐ No ☐ Unsure

Comments (optional):

**DAY 1, SESSION 3: Country Team Panel A – Day 1, 10:45-12:30**

*On a scale of 1-5, please evaluate this session by circling your answer*

Usefulness of the material

Poor…………………..…Fair……………..…...…Average……………….…...Good…………..…Excellent

1 2 3 4 5

Organization of session

Poor…………………..…Fair……………..…...…Average……………….…...Good…………..…Excellent

1 2 3 4 5

Clarity of presentation

Poor…………………..…Fair……………..…...…Average……………….…...Good…………..…Excellent

1 2 3 4 5

Would you recommend that this session be included in future PEPFAR policy monitoring workshops with other countries? Check one.

☐ Yes ☐ No ☐ Unsure

Comments (optional):

PF/PFIP Workshop (1A)

**DAY 1, SESSION 4: Country Team Panel B – 1:30-3:15**

*On a scale of 1-5, please evaluate this session by circling your answer*

Usefulness of the material

Poor…………………..…Fair……………..…...…Average……………….…...Good…………..…Excellent

1 2 3 4 5

Organization of session

Poor…………………..…Fair……………..…...…Average……………….…...Good…………..…Excellent

1 2 3 4 5

Clarity of presentation

Poor…………………..…Fair……………..…...…Average……………….…...Good…………..…Excellent

1 2 3 4 5

Would you recommend that this session be included in future PEPFAR policy monitoring workshops with other countries? Check one.

☐ Yes ☐ No ☐ Unsure

Comments (optional):

**DAY 1, SESSION 5: The Policy Process – 3:30-4:45**

*On a scale of 1-5, please evaluate this session by circling your answer*

Usefulness of the material

Poor…………………..…Fair……………..…...…Average……………….…...Good…………..…Excellent

1 2 3 4 5

Organization of session

Poor…………………..…Fair……………..…...…Average……………….…...Good…………..…Excellent

1 2 3 4 5

Clarity of presentation

Poor…………………..…Fair……………..…...…Average……………….…...Good…………..…Excellent

1 2 3 4 5

Would you recommend that this session be included in future PEPFAR policy monitoring workshops with other countries? Check one.

☐ Yes ☐ No ☐ Unsure

Comments (optional):

**DAY 2, SESSION 1: Overview of Roadmap – 9:00–10:00**

*On a scale of 1-5, please evaluate this session by circling your answer*

Usefulness of the material

Poor…………………..…Fair……………..…...…Average……………….…...Good…………..…Excellent

1 2 3 4 5

Organization of session

Poor…………………..…Fair……………..…...…Average……………….…...Good…………..…Excellent

1 2 3 4 5

Clarity of presentation

Poor…………………..…Fair……………..…...…Average……………….…...Good…………..…Excellent

1 2 3 4 5

Would you recommend that this session be included in future PEPFAR policy monitoring workshops with other countries? Check one.

☐ Yes ☐ No ☐ Unsure

Comments (optional):

PF/PFIP Workshop (1A)

**DAY 2, SESSION 2: Framework for Monitoring Policy Process – 10:00–10:45**

*On a scale of 1-5, please evaluate this session by circling your answer*

Usefulness of the material

Poor…………………..…Fair……………..…...…Average……………….…...Good…………..…Excellent

1 2 3 4 5

Organization of session

Poor…………………..…Fair……………..…...…Average……………….…...Good…………..…Excellent

1 2 3 4 5

Clarity of presentation

Poor…………………..…Fair……………..…...…Average……………….…...Good…………..…Excellent

1 2 3 4 5

Would you recommend that this session be included in future PEPFAR policy monitoring workshops with other countries? Check one.

☐ Yes ☐ No ☐ Unsure

Comments (optional):

**DAY 2, SESSION 3: Country Team Discussion A – 11:00-1:00**

*On a scale of 1-5, please evaluate this session by circling your answer*

Usefulness of the material

Poor…………………..…Fair……………..…...…Average……………….…...Good…………..…Excellent

1 2 3 4 5

Organization of session

Poor…………………..…Fair……………..…...…Average……………….…...Good…………..…Excellent

1 2 3 4 5

Clarity of presentation

Poor…………………..…Fair……………..…...…Average……………….…...Good…………..…Excellent

1 2 3 4 5

Would you recommend that this session be included in future PEPFAR policy monitoring workshops with other countries? Check one.

☐ Yes ☐ No ☐ Unsure

Comments (optional):

PF/PFIP Workshop (1A)

**DAY 2, SESSION 4: Framework for Monitoring and Evaluation – 2:00-3:00**

*On a scale of 1-5, please evaluate this session by circling your answer*

Usefulness of the material

Poor…………………..…Fair……………..…...…Average……………….…...Good…………..…Excellent

1 2 3 4 5

Organization of session

Poor…………………..…Fair……………..…...…Average……………….…...Good…………..…Excellent

1 2 3 4 5

Clarity of presentation

Poor…………………..…Fair……………..…...…Average……………….…...Good…………..…Excellent

1 2 3 4 5

Would you recommend that this session be included in future PEPFAR policy monitoring workshops with other countries? Check one.

☐ Yes ☐ No ☐ Unsure

Comments (optional):

**DAY 2, SESSION 5: Country Team Discussion B – 3:00-5:00**

*On a scale of 1-5, please evaluate this session by circling your answer*

Usefulness of the material

Poor…………………..…Fair……………..…...…Average……………….…...Good…………..…Excellent

1 2 3 4 5

Organization of session

Poor…………………..…Fair……………..…...…Average……………….…...Good…………..…Excellent

1 2 3 4 5

Clarity of presentation

Poor…………………..…Fair……………..…...…Average……………….…...Good…………..…Excellent

1 2 3 4 5

Would you recommend that this session be included in future PEPFAR policy monitoring workshops with other countries? Check one.

☐ Yes ☐ No ☐ Unsure

Comments (optional):

**DAY 3, SESSION 1: Policy objectives in the Global Context – 9:00–10:00**

*On a scale of 1-5, please evaluate this session by circling your answer*

Usefulness of the material

Poor…………………..…Fair……………..…...…Average……………….…...Good…………..…Excellent

1 2 3 4 5

Organization of session

Poor…………………..…Fair……………..…...…Average……………….…...Good…………..…Excellent

1 2 3 4 5

Clarity of presentation

Poor…………………..…Fair……………..…...…Average……………….…...Good…………..…Excellent

1 2 3 4 5

Would you recommend that this session be included in future PEPFAR policy monitoring workshops with other countries? Check one.

☐ Yes ☐ No ☐ Unsure

Comments (optional):

PF/PFIP Workshop (1A)

**DAY 3, SESSION 2: Overview of methodologies and tools – 10:00-10:30**

*On a scale of 1-5, please evaluate this session by circling your answer*

Usefulness of the material

Poor…………………..…Fair……………..…...…Average……………….…...Good…………..…Excellent

1 2 3 4 5

Organization of session

Poor…………………..…Fair……………..…...…Average……………….…...Good…………..…Excellent

1 2 3 4 5

Clarity of presentation

Poor…………………..…Fair……………..…...…Average……………….…...Good…………..…Excellent

1 2 3 4 5

Would you recommend that this session be included in future PEPFAR policy monitoring workshops with other countries? Check one.

☐ Yes ☐ No ☐ Unsure

Comments (optional):

**DAY 3, SESSION 3: Policy monitoring tools and approaches – 10:45-11:30**

**Title: ______________________________________________________________________**

*On a scale of 1-5, please evaluate this session by circling your answer*

Usefulness of the material

Poor…………………..…Fair……………..…...…Average……………….…...Good…………..…Excellent

1 2 3 4 5

Organization of session

Poor…………………..…Fair……………..…...…Average……………….…...Good…………..…Excellent

1 2 3 4 5

Clarity of presentation

Poor…………………..…Fair……………..…...…Average……………….…...Good…………..…Excellent

1 2 3 4 5

Would you recommend that this session be included in future PEPFAR policy monitoring workshops with other countries? Check one.

☐ Yes ☐ No ☐ Unsure

Comments (optional):

PF/PFIP Workshop (1A)

**DAY 3, SESSION 4: Policy monitoring tools and approaches – 11:30-12:30**

**Title: ______________________________________________________________________**

*On a scale of 1-5, please evaluate this session by circling your answer*

Usefulness of the material

Poor…………………..…Fair……………..…...…Average……………….…...Good…………..…Excellent

1 2 3 4 5

Organization of session

Poor…………………..…Fair……………..…...…Average……………….…...Good…………..…Excellent

1 2 3 4 5

Clarity of presentation

Poor…………………..…Fair……………..…...…Average……………….…...Good…………..…Excellent

1 2 3 4 5

Would you recommend that this session be included in future PEPFAR policy monitoring workshops with other countries? Check one.

☐ Yes ☐ No ☐ Unsure

Comments (optional):

**DAY 3, LUNCH: Legal mechanisms to address HIV – 12:30-1:30**

*On a scale of 1-5, please evaluate this session by circling your answer*

Usefulness of the material

Poor…………………..…Fair……………..…...…Average……………….…...Good…………..…Excellent

1 2 3 4 5

Organization of session

Poor…………………..…Fair……………..…...…Average……………….…...Good…………..…Excellent

1 2 3 4 5

Clarity of presentation

Poor…………………..…Fair……………..…...…Average……………….…...Good…………..…Excellent

1 2 3 4 5

Would you recommend that this session be included in future PEPFAR policy monitoring workshops with other countries? Check one.

☐ Yes ☐ No ☐ Unsure

Comments (optional):

PF/PFIP Workshop (1A)

**DAY 3, SESSION 5: Country Team work to develop Road Map A – 1:30-2:30**

*On a scale of 1-5, please evaluate this session by circling your answer*

Usefulness of the material

Poor…………………..…Fair……………..…...…Average……………….…...Good…………..…Excellent

1 2 3 4 5

Organization of session

Poor…………………..…Fair……………..…...…Average……………….…...Good…………..…Excellent

1 2 3 4 5

Clarity of presentation

Poor…………………..…Fair……………..…...…Average……………….…...Good…………..…Excellent

1 2 3 4 5

Would you recommend that this session be included in future PEPFAR policy monitoring workshops with other countries? Check one.

☐ Yes ☐ No ☐ Unsure

Comments (optional):

**DAY 3, SESSION 6: Closing the Feedback Loop – 2:30-3:15**

*On a scale of 1-5, please evaluate this session by circling your answer*

Usefulness of the material

Poor…………………..…Fair……………..…...…Average……………….…...Good…………..…Excellent

1 2 3 4 5

Organization of session

Poor…………………..…Fair……………..…...…Average……………….…...Good…………..…Excellent

1 2 3 4 5

Clarity of presentation

Poor…………………..…Fair……………..…...…Average……………….…...Good…………..…Excellent

1 2 3 4 5

Would you recommend that this session be included in future PEPFAR policy monitoring workshops with other countries? Check one.

☐ Yes ☐ No ☐ Unsure

Comments (optional):

PF/PFIP Workshop (1A)

**DAY 3, SESSION 7: Country Team work to develop Road Map B – 3:30-4:45**

*On a scale of 1-5, please evaluate this session by circling your answer*

Usefulness of the material

Poor…………………..…Fair……………..…...…Average……………….…...Good…………..…Excellent

1 2 3 4 5

Organization of session

Poor…………………..…Fair……………..…...…Average……………….…...Good…………..…Excellent

1 2 3 4 5

Clarity of presentation

Poor…………………..…Fair……………..…...…Average……………….…...Good…………..…Excellent

1 2 3 4 5

Would you recommend that this session be included in future PEPFAR policy monitoring workshops with other countries? Check one.

☐ Yes ☐ No ☐ Unsure

Comments (optional):

**DAY 4, SESSION 1: Discussion of Progress and Problem Solving – 9:00–9:30**

*On a scale of 1-5, please evaluate this session by circling your answer*

Usefulness of the material

Poor…………………..…Fair……………..…...…Average……………….…...Good…………..…Excellent

1 2 3 4 5

Organization of session

Poor…………………..…Fair……………..…...…Average……………….…...Good…………..…Excellent

1 2 3 4 5

Clarity of presentation

Poor…………………..…Fair……………..…...…Average……………….…...Good…………..…Excellent

1 2 3 4 5

Would you recommend that this session be included in future PEPFAR policy monitoring workshops with other countries? Check one.

☐ Yes ☐ No ☐ Unsure

Comments (optional):

PF/PFIP Workshop (1A)

**DAY 4, SESSION 2: Best Practices in Policy Implementation – 9:30-10:30**

*On a scale of 1-5, please evaluate this session by circling your answer*

Usefulness of the material

Poor…………………..…Fair……………..…...…Average……………….…...Good…………..…Excellent

1 2 3 4 5

Organization of session

Poor…………………..…Fair……………..…...…Average……………….…...Good…………..…Excellent

1 2 3 4 5

Clarity of presentation

Poor…………………..…Fair……………..…...…Average……………….…...Good…………..…Excellent

1 2 3 4 5

Would you recommend that this session be included in future PEPFAR policy monitoring workshops with other countries? Check one.

☐ Yes ☐ No ☐ Unsure

Comments (optional):

**DAY 4, SESSION 3: Assessing capacity – 10:45-12:00**

*On a scale of 1-5, please evaluate this session by circling your answer*

Usefulness of the material

Poor…………………..…Fair……………..…...…Average……………….…...Good…………..…Excellent

1 2 3 4 5

Organization of session

Poor…………………..…Fair……………..…...…Average……………….…...Good…………..…Excellent

1 2 3 4 5

Clarity of presentation

Poor…………………..…Fair……………..…...…Average……………….…...Good…………..…Excellent

1 2 3 4 5

Would you recommend that this session be included in future PEPFAR policy monitoring workshops with other countries? Check one.

☐ Yes ☐ No ☐ Unsure

Comments (optional):

PF/PFIP Workshop (1A)

**DAY 4, SESSION 4: Country Team work to develop Road Map C – 12:00-1:00**

*On a scale of 1-5, please evaluate this session by circling your answer*

Usefulness of the material

Poor…………………..…Fair……………..…...…Average……………….…...Good…………..…Excellent

1 2 3 4 5

Organization of session

Poor…………………..…Fair……………..…...…Average……………….…...Good…………..…Excellent

1 2 3 4 5

Clarity of presentation

Poor…………………..…Fair……………..…...…Average……………….…...Good…………..…Excellent

1 2 3 4 5

Would you recommend that this session be included in future PEPFAR policy monitoring workshops with other countries? Check one.

☐ Yes ☐ No ☐ Unsure

Comments (optional):

**DAY 4, SESSION 5: Post-Workshop Plan – 2:00-3:00**

*On a scale of 1-5, please evaluate this session by circling your answer*

Usefulness of the material

Poor…………………..…Fair……………..…...…Average……………….…...Good…………..…Excellent

1 2 3 4 5

Organization of session

Poor…………………..…Fair……………..…...…Average……………….…...Good…………..…Excellent

1 2 3 4 5

Clarity of presentation

Poor…………………..…Fair……………..…...…Average……………….…...Good…………..…Excellent

1 2 3 4 5

Would you recommend that this session be included in future PEPFAR policy monitoring workshops with other countries? Check one.

☐ Yes ☐ No ☐ Unsure

Comments (optional):

PF/PFIP Workshop (1A)

**DAY 4, SESSION 6: Country Team Presentations – 3:15-4:30**

*On a scale of 1-5, please evaluate this session by circling your answer*

Usefulness of the material

Poor…………………..…Fair……………..…...…Average……………….…...Good…………..…Excellent

1 2 3 4 5

Organization of session

Poor…………………..…Fair……………..…...…Average……………….…...Good…………..…Excellent

1 2 3 4 5

Clarity of presentation

Poor…………………..…Fair……………..…...…Average……………….…...Good…………..…Excellent

1 2 3 4 5

Would you recommend that this session be included in future PEPFAR policy monitoring workshops with other countries? Check one.

☐ Yes ☐ No ☐ Unsure

Comments (optional):
