# Supplementary material for: Monitoring HIV and AIDS Related Policy Reforms: A Road Map to Strengthen Policy Monitoring and Implementation in PEPFAR Partner Countries
Source: PLoS One. 2016 Feb 25;11(2):e0146720. doi: 10.1371/journal.pone.0146720 (PMC4767332; doi:10.1371/journal.pone.0146720)
Supplement: S4 File — (DOCX) [file pone.0146720.s004.docx]

**Partnership Framework Policy Monitoring Workshop Day 1 Feedback**

1.) Did the day one session material meet your expectations? Check one.

☐ Yes ☐ No ☐ Unsure

Please explain.

2.) What do you think worked well during Day 1?

Please explain.

3.) What do you think did not work so well during Day 1?

Please explain.

4.) Do you feel confident in applying what you’ve learned during Day 1 in your country?

Please explain.

5.) Do you think you will have the opportunity to apply what you’ve learned during the next 6 months?

☐ Yes ☐ No ☐ Unsure

Please explain.

**Partnership Framework Policy Monitoring Workshop Day 2 Feedback**

1.) Did the day one session material meet your expectations? Check one.

☐ Yes ☐ No ☐ Unsure

Please explain.

2.) What do you think worked well during Day 2?

Please explain.

3.) What do you think did not work so well during Day 2?

Please explain.

4.) Do you feel confident in applying what you’ve learned during Day 2 in your country?

Please explain.

5.) Do you think you will have the opportunity to apply what you’ve learned during the next 6 months?

☐ Yes ☐ No ☐ Unsure

Please explain.

**Partnership Framework Policy Monitoring Workshop Day 3 Feedback**

1.) Did the day one session material meet your expectations? Check one.

☐ Yes ☐ No ☐ Unsure

Please explain.

2.) What do you think worked well during Day 3?

Please explain.

3.) What do you think did not work so well during Day 3?

Please explain.

4.) Do you feel confident in applying what you’ve learned during Day 3 in your country?

Please explain.

5.) Do you think you will have the opportunity to apply what you’ve learned during the next 6 months?

☐ Yes ☐ No ☐ Unsure

Please explain.

**Partnership Framework Policy Monitoring Workshop Day 4 Feedback**

1.) Did the day one session material meet your expectations? Check one.

☐ Yes ☐ No ☐ Unsure

Please explain.

2.) What do you think worked well during Day 4?

Please explain.

3.) What do you think did not work so well during Day 4?

Please explain.

4.) Do you feel confident in applying what you’ve learned during Day 4 in your country?

Please explain.

5.) Do you think you will have the opportunity to apply what you’ve learned during the next 6 months?

☐ Yes ☐ No ☐ Unsure

Please explain.
