# Supplementary material for: Monitoring HIV and AIDS Related Policy Reforms: A Road Map to Strengthen Policy Monitoring and Implementation in PEPFAR Partner Countries
Source: PLoS One. 2016 Feb 25;11(2):e0146720. doi: 10.1371/journal.pone.0146720 (PMC4767332; doi:10.1371/journal.pone.0146720)
Supplement: S5 File — (DOCX) [file pone.0146720.s005.docx]

**Partnership Framework Policy Monitoring Workshop**

**DISCUSSION GROUP – DAY 4, 12:00 – 1:00**

OVERVIEW: The discussion group is intended to investigate the effectiveness of the workshop and obtain ideas for improving future such workshops. Participation in these discussion groups is entirely **voluntary.**

- Three separate sessions (USG, country government, CSO representatives)
- One facilitator and two note takers per session

1. To what extent did the workshop meet your expectations? If so, in what ways?

Prompts

- What has been the most useful part of the workshop?
- To what extent were you able to share with and learn from the experiences of the other countries participating in the workshop?
- Did the workshop help increase your knowledge of how policy affects health?
- Is your team better prepared for monitoring PF/PFIP policy objectives? If so, how?
- Are there other ways your country team benefited from the workshop?

1. To what extent did the workshop fail to meet your expectations?

Prompts

- What has been the least useful part of the workshop?
- What challenges did your team face while at the workshop?

1. To what extent were the different workshop modes (didactic sessions, small group discussions, etc.) effective in helping your team learn and share experiences with other country teams?
2. Do you have a good sense of what your team needs to do next?
3. How can we improve our workshop approach or plan in order to better serve country teams in their policy monitoring activities?

Prompts

- - What ideas do you have about how we can better clarify the concepts of policy objective, policy development, policy process, and policy monitoring?
  - How can we improve the agenda for future policy monitoring workshops?

Follow-up: The facilitator should also ask the participants’ consent to be contacted in 2, 6 and 12 months to continue the evaluation of the workshop.
