# Supplementary material for: Monitoring HIV and AIDS Related Policy Reforms: A Road Map to Strengthen Policy Monitoring and Implementation in PEPFAR Partner Countries
Source: PLoS One. 2016 Feb 25;11(2):e0146720. doi: 10.1371/journal.pone.0146720 (PMC4767332; doi:10.1371/journal.pone.0146720)
Supplement: S6 File — (DOCX) [file pone.0146720.s006.docx]

**PEPFAR Partnership Frameworks Policy Monitoring Workshops**

**3 Month Evaluation Questions**

**Administered via Anonymous Online Survey**

1. Your Country:
2. Your Sector:
3. On a scale of 1 to 5 (1 being least useful and 5 being most useful), how would you rate the usefulness of the PEPFAR Partnership Framework Policy Monitoring Workshop in helping you conduct policy monitoring
4. Are you better prepared to monitor policy activities after attending the workshop in Dar es Salaam?
5. Please explain your answer to the previous question (question 4).
6. What additional information, tools, or technical assistance do you need to effectively monitor policies?
7. Have you and/or your country team implemented or further refined the action plan developed during the Policy Monitoring workshop in Dar es Salaam?
8. Please provide specific examples of how you have implemented or further developed this plan or why you haven't.
9. Any other comments or thoughts?
